# Tergal and pleural structures contribute to the formation of ectopic prothoracic wings in cockroaches

**DOI:** 10.1098/rsos.160347

**Published:** 2016-08-03

**Authors:** Moysés Elias-Neto, Xavier Belles

**Affiliations:** 1 Institute of Evolutionary Biology, CSIC-Universitat Pompeu Fabra, Passeig Marítim de la Barceloneta 37, 08003 Barcelona, Spain; 2 Departamento de Biologia, Faculdade de Filosofia, Ciências e Letras de Ribeirão Preto, Universidade de São Paulo, Av. Bandeirantes 3900, 14040-901 Ribeirão Preto, SP, Brazil

**Keywords:** origin insect wings, sex combs reduced, *Blattella*, *Drosophila*, *Tribolium*

## Abstract

Wings were a fundamental morphological innovation for the adaptive radiation of insects, the most diversified group among all animals. Pterygote insects have two pairs of wings, the mesothoracic (T2) forewings and the metathoracic (T3) hindwings, whereas the prothorax (T1) is wingless. Using RNA interference approaches, we have found that the gene *Sex combs reduced* (*Scr*) determines the wingless identity of T1 in the cockroach *Blattella germanica*. Interference of *Scr* triggers the formation of ectopic wing structures in T1, which are formed from the expansion of the latero-posterior region of the pronotum, along with a contribution of the epimeron, a pleurite of T1. These data support the theory of a dual origin for insect wings, from pronotal (tergal origin theory) and pleural (pleural origin theory) structures and genes.

## 1. Introduction

Wings were a morpho-functional innovation crucial for the spectacular radiation of insects, the most speciose lineage among all animals. Present winged insects, the Pterygota, have two pairs of wings located on the second (T2) and third (T3) thoracic segments, whereas the first thoracic segment (T1) is wingless [1]. Wing morphogenesis in T2 and T3 is regulated by a complex gene cascade [2], whereas the wingless identity of T1 mainly results from the action of the Hox gene *Sex combs reduced* (*Scr*). The first data suggesting that *Scr* might repress wing formation in T1 came from the work of Carroll *et al*. [3], who showed that it inhibits the expression of *vestigial* (*vg*) and *snail* (two genes required for wing formation) in the primordia of dorsal discs of the fruit fly, *Drosophila melanogaster*. Two years later, Rogers *et al*. [4] reported that *D. melanogaster* carrying a hypomorphic heterozygous allele with a null allele for *Scr* formed wing tissue in T1. Similar experiments carried out with the *Scr* orthologue *Cephalothorax* in the flour beetle, *Tribolium castaneum*, elicited the formation of elytra in T1 [5], a phenotype that was also observed when depleting *Scr* expression by RNA interference (RNAi) [6]. More recently, formation of wing-like tissue in T1 after RNAi of *Scr* has been reported in the large milkweed bug, *Oncopeltus fasciatus* [7,8], and the American cockroach, *Periplaneta americana* [9].

The origin of insect wings has been the object of continuous interest (and considerable controversy), and has led to two basic theories (see [10] for a recent review). One theory proposes that wings originated from an extension of the thoracic tergum, first forming paranotal lobes and then fully articulated wings. This tergal origin theory was initially suggested by F. Müller in 1875, formalized by Crampton [11] and supported by Snodgrass [12] and Hamilton [13] (see [14]). The theory of tergal origin has also been invoked in modern developmental studies based on molecular approaches [9,15].

The second theory considers the wing is derived from pleural structures and also has very ancient origins, dating back to Oken [16] and Woodworth [17]. In modern times, it has been championed by Kukalová-Peck [18–21], who proposed the wing derives from articulated exites that were located on the proximal leg segments of ancestral insects that migrated dorsally and finally formed the articulated and flying wing. An influential paper by Averof & Cohen [22] reported that insect wing-related genes are expressed in dorsal gills of crustacean branched limbs, providing support for the pleural origin theory. Recent morphological and molecular investigations have shown that the embryonic subcoxa contribute to form pleural sclerites of the adult [23] and have also provided significant arguments in favour of the pleural origin theory.

In the past, the theory of tergal origin has proved the most popular, possibly because it appears coherent with the flat form and position of modern wings; however, it does not account for the complex musculature and articulations needed for flight functions. Conversely, the theory of pleural origin explains the origin of muscles and articulations as they are already contained in the leg branch limb, but this hypothesis is counterintuitive when trying to imagine how a leg branch can transform into a flattened and flexible structure like the wing of a flying insect. A possible solution to this disparity might be to merge both theories, thus explaining the origin of wings according to a dual contribution of tergal and pleural structures. Indeed this eclectic approach has a historical background as it was first suggested by Crampton [11], although he clearly favoured the tergal origin theory. More recently, Rasnitsyn [24] modified the tergal origin theory of wings by adding the contribution of proximal leg segments. However, the most convincing data pointing to wings having a dual origin, combining tergal and pleural contributions, have been produced recently following molecular approaches by the groups of Hayashi and co-workers [25] and Tomoyasu and co-workers [26].

The Hayashi group used the bristletail *Pedetontus unimaculatus* (apterygota, Archaeognatha) and the mayfly *Ephoron eophilum* (Paleoptera, Ephemeroptera) as experimental subjects (both of which develop dorsal limb branches, styli and tracheal gills, respectively) and studied the expression of three genes key to insect wing development, *wingless* (*wg*), *vestigial* (*vg*) and *apterous* (*ap*). Results showed that *wg* and *vg* are expressed in the primordia of styli and tracheal gills, whereas the lateral tergal margin expresses these two genes in addition to *ap*. The authors concluded that insect wings might have originated from the fusion of the modules corresponding to the tergal margin and to the limb branch [25].

The Tomoyasu research group used *T. castaneum* (Endopterygota, Coleoptera) as model and RNAi experiments, and found that the lateral tergal margin (designated by these authors as the carinated margin) as well as some pleural plates in T1 are *vg* dependent [26]. Interestingly, in T2 and T3 *vg* functions in the wings, and systemic interference of *Scr*, a gene that determines the wingless identity of T1, triggers the homeotic transformation of T1 into T2 including the formation of ectopic wings in T1 through the contribution of those T1 structures where *vg* is expressed [6,26]. These data provide compelling support for the theory that incorporates a dual origin of wings, with tergal and pleural contributions.

A most recent paper by Popadić and co-workers, using the hemimetabolan species, *O. fasciatus* (Condylognatha, Hemiptera) as model, reports that the T1 wing structures formed after *Scr* depletion in this species are mainly of dorsal origin, although gene expression differences between the ectopic T1 wing structure and T2 and T3 wings suggest that ventral structures also contribute to wing formation [8]. These results would also support, thus, a dual origin for insect wings.

This work aims to provide a contribution in this context by studying the changes on T1 triggered by *Scr* depletion using the German cockroach *Blattella germanica* as the experimental subject. *Blattella germanica* is a hemimetabolan species like *O. fasciatus*, but belongs to the subclass Polyneoptera, the sister group of Condylognatha + Psocodea + Endopterygota (=Holometabola) [27].

## 2. Material and methods

### 2.1. Insects

*Blattella germanica* specimens used in the experiments were obtained from a colony reared in the dark at 30 ± 1°C and 60–70% RH. They were carbon dioxide-anaesthetized prior to dissections and tissue sampling.

### 2.2. RNA extraction and retrotranscription to cDNA

All RNA extractions were carried out with the Gen Elute Mammalian Total RNA kit (Sigma-Aldrich, Madrid, Spain). An amount of 400 ng from each RNA extraction was treated with DNase (Promega, Madison, WI, USA) and reverse transcribed with Superscript II reverse transcriptase (Invitrogen, Carlsbad, CA, USA) and random hexamers (Promega). RNA quantity and quality were estimated by spectrophotometric absorption at 260 nm in a Nanodrop spectrophotometer ND-1000^®^ (NanoDrop Technologies, Wilmington, DE, USA).

### 2.3. Determination of mRNA levels with quantitative real-time PCR

Quantitative real-time PCR (qRT-PCR) reactions were carried out in triplicate in an iQ5 real-time PCR detection system (Bio-Rad Laboratories, Madrid, Spain), using SYBR®Green (Power SYBR® Green PCR Master Mix; Applied Biosystems, Madrid, Spain). A control without template was included in all batches. In addition to measuring mRNA levels of *Scr*, we also measured the expression of the following 16 genes involved in wing formation, whose sequence was available in the *B. germanica* transcriptome of metanotum epidermis obtained in our laboratory (GEO accession number GSM1569374): *apterous a* (*ap-a*), *blistered* (*bs*), *cut* (*ct*), *delta* (*Dl*), *decapentaplegic* (*dpp*), *epidermal growth factor receptor* (*Egfr*), *engrailed* (*en*), *notch* (*N*), *nubbin* (*nub*), *rhomboid* (*rho*), *serrate* (*Ser*), *scalloped* (*sd*), *spalt* (*salm*), *ultrabithorax* (*Ubx*), *vestigial* (*vg*) and *wingless* (*wg*). The primers used for each transcript measured are detailed in the electronic supplementary material, table S1. The efficiency of each primer set was first validated by constructing a standard curve through four serial dilutions. mRNA levels were quantified relative to BgActin-5c expression if not stated otherwise, using the Bio-Rad iQ5 Standard Edition Optical System Software (v. 2.0); primers are indicated in the electronic supplementary material table S1. We followed a method based on Ct (threshold-cycle) according to the Pfaffl mathematical model [28], simplifying to 2^ΔΔCt^ because the calculated efficiency values for studied genes and BgActin-5c amplicons were always within the range of 95–100%; therefore, no correction for efficiency was used in further calculations. Results are given as copies of mRNA per 1000 copies of BgActin-5c mRNA.

### 2.4. RNA interference

In general, we followed the RNAi approach previously described [29]. The primers to generate the templates for the dsRNA targeting *Scr* (dsScr) are indicated in the electronic supplementary material, table S1. The fragments were amplified by PCR and cloned into the pSTBlue-1 vector (Novagen, Madrid, Spain). In all cases, we used a 307 bp sequence from *Autographa californica* nucleopolyhydrosis virus (accession no. K01149, from nucleotide 370 to 676) as control dsRNA (dsMock). The dsRNAs were prepared as reported elsewhere [29]. Female nymphs were treated with three 2 µg doses of dsScr in 1 µl volume injected into the abdomen with a 5 µl Hamilton microsyringe, the first in freshly emerged fourth instar, the second in freshly emerged fifth instar and the third in freshly emerged sixth instar (*n *= 51). Control specimens were equivalently treated with dsMock (*n *= 23). Transcript decrease was examined by qRT-PCR in T1 on day 6 of the sixth nymphal instar.

### 2.5. Morphological studies

Routine examinations and photographs were made with an optic stereomicroscope Zeiss DiscoveryV8. For scanning electron microscopy (SEM), the specimens were dried at room temperature and glued to stubs with colloidal silver, sputter coated with gold palladium, and examined with a Hitachi S-3500N (Nissei Sangyo Co. Ltd., Tokyo, Japan) SEM operating at 5 kV. Integument preparations were briefly rinsed with Ringer saline (NaCl 0.17 M; KCl 0.01 M; CaCl_2_ 0.003 M), and kept for 12 h in cold (4°C) fixative (4% paraformaldehyde in 0.1 M phosphate buffer, pH 7.3). Next, integuments were dehydrated in a graded ethanol series (70%, 80%, 90% and 95% ethanol in water, v/v) for 15 min each, followed by two rinses in 100% for 15 min each. Samples were critical point-dried in liquid CO_2_ using a BAL-TEC CPD 030 critical point drying apparatus (Balzers Union, Balzers, Germany).

### 2.6. Statistics

Quantitative data are expressed as mean ± s.e.m. In qRT-PCR determinations, statistical analyses between groups were tested by the REST 2008 program (Relative Expression Software Tool V 2.0.7; Corbett Research) [28]. This program makes no assumptions about the distributions, evaluating the significance of the derived results by pair-wise fixed reallocation randomization test tool.

## 3. Results

**Figure 1.**
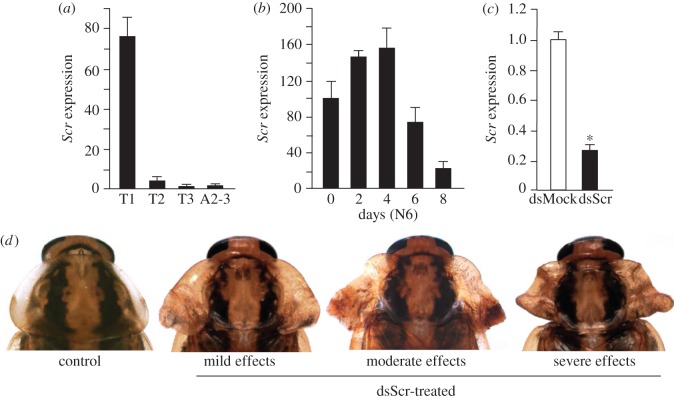
Expression of *Scr* in *Blattella germanica* and RNAi effects. (*a*) mRNA levels of *Scr* in the three thoracic segments, prothorax (T1), mesothorax (T2) and metathorax (T3), and in the 2nd and 3rd abdominal segments (A2–3) of female nymphs, measured on day 6 of sixth (last) instar (N6D6). (*b*) mRNA levels of *Scr* during N6 in T1. (*c*) mRNA levels of *Scr* measured in T1 on N6D6 in controls (dsMock-treated) and dsScr-treated insects; female nymphs were treated with three 2 µg doses of dsRNA, the first injected in freshly emerged fourth instar (N4D0), the second in N5D0 and the third in N6D0. (*d*) Dorsal view of the pronotum of adult females from the control (dsMock-treated, *n* = 23) and dsScr-treated groups (*n* = 51), including examples of mild, moderate and severe effects. Expression (*a*–*c*) is represented as mRNA copies per 1000 copies of actin-5c (mean ± s.e.m., *n* = 4). In (*c*), expression is normalized against respective control values (indicated as 1) and the asterisk indicates statistically significant differences with respect to controls (*p* < 0.05), according to REST [28].

*Scr* is significantly expressed only in T1 (figure 1*a*). In the last instar nymph, expression follows a pattern of gradual increase until day 4, and then a rapid decrease during days 6–8 (figure 1*b*). We depleted *Scr* mRNA levels in female nymphs following an RNAi approach, applying three 2 µg doses of dsScr (treated) or dsMock (controls), the first injected in freshly emerged fourth instar (N4D0), the second in N5D0 and the third in N6D0. Transcript decrease was examined in T1 on N6D6, when the moulting peak of ecdysone is being produced [30] and wing maturation proceeds in T2 and T3 [31]. Results showed that RNAi efficiently reduced *Scr* expression (approx. 75% *Scr* mRNA reduction; figure 1*c*).

**Figure 2.**
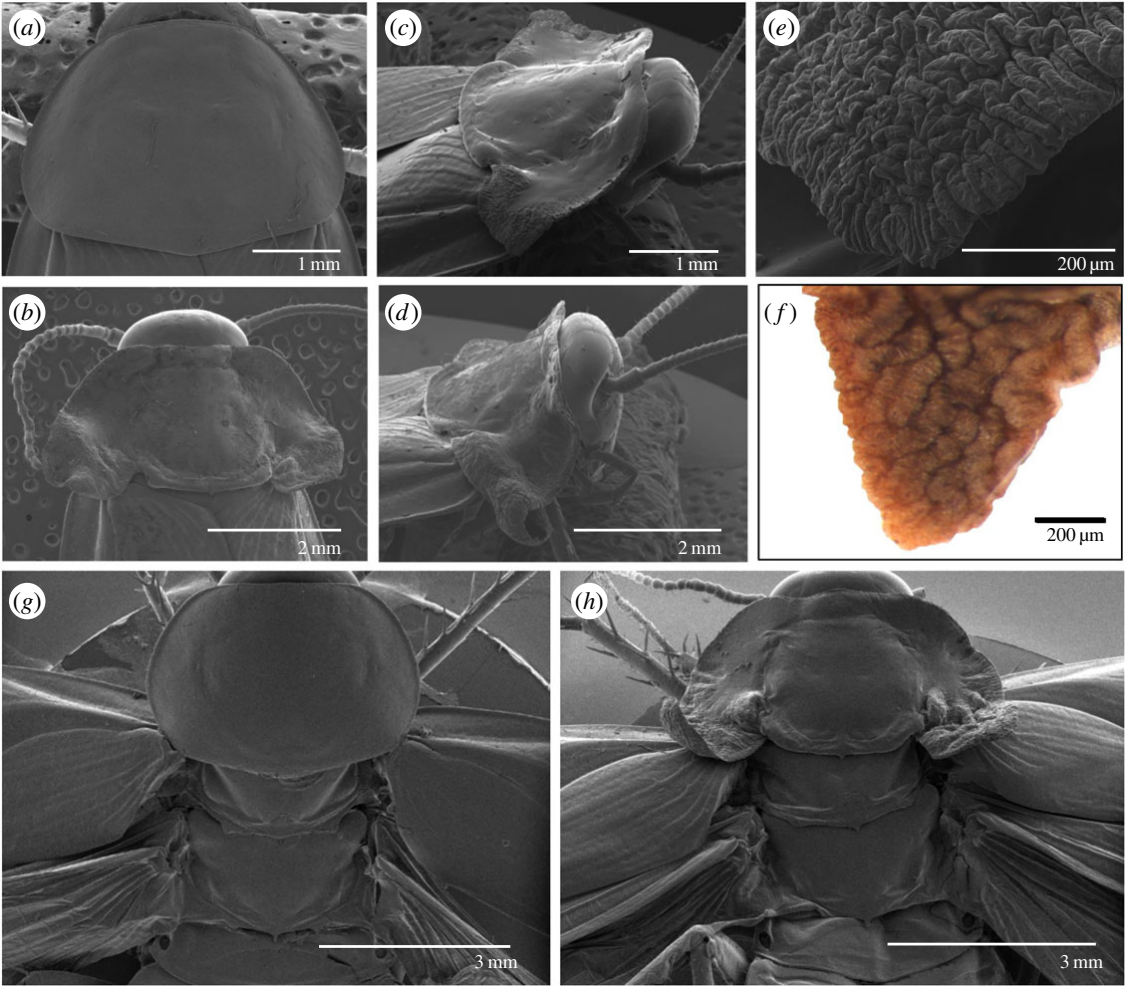
SEM examinations of the effects of *Scr* depletion in *Blattella germanica* on the thorax dorsal region. (*a*) Dorsal view of T1 of a control adult. (*b*) Dorsal view of T1 of an adult obtained after dsScr treatments, with moderately developed lateral expansions (moderate phenotype). (*c*) Dorso-lateral view of T1 of an adult obtained after dsScr treatments, with moderately developed lateral expansions (moderate phenotype). (*d*) Dorso-lateral view of T1 of an adult obtained after dsScr treatments, with conspicuously developed lateral expansions (severe phenotype). (*e*) Detail of the tip of the right lateral expansion shown in (*c*). (*f*) Tip of the wing bud located in the pocket of T2 lateral expansions on day 6 of the last nymphal instar. (*g*,*h*) Dorsal view of T1, T2 and T3 of a control adult (*g*) and an adult obtained following dsScr treatments (*h*) with the T2 and T3 wings extended.

DsScr-treated nymphs (*n* = 51) moulted to N5, to N6 and then to adults as in dsMock-treated controls (*n* = 23). However, the adults emerging from dsScr-treated nymphs showed conspicuous latero-posterior expansions of the pronotal edge, from mild to moderate or severe (figure 1*d*). In mild phenotypes, the pronotum presented a similar shape to the controls, although slightly more transverse (in controls, the pronotum was approximately as long as it was wide, figures  1*d* and 2*a*), with the latero-posterior region slightly expanded, especially near the base, forming a lobular structure with a slightly wrinkled surface (figure 1*d*). Moderate phenotypes had a clearly transverse pronotum, the latero-posterior region was more expanded, and the lobular basal structure notably wrinkled (figure 1*d*). Moreover, the pronotum, which was uniformly convex in the controls (figures  1*d* and 2*a*), showed two longitudinal grooves separating the actual pronotal disc from the latero-posterior expansions, which ended at the base with respective notches. The anterior and posterior edges each revealed very marked transverse grooves delimiting the central part of the pronotal disc, whose length was greater than its width and was almost flat. Finally, in the centre of the posterior edge, there was a small, backward-facing, triangular protrusion (figures  1*d* and 2*b*,*c*). The specimens showing the most severe effects were essentially similar, but their latero-posterior expansions were even more developed, especially the lobular basal structure, which was larger, heavily wrinkled and folded, and adopted curled morphologies (figures  1*d* and 2*d*). At high magnification, the wrinkled lobular structure of these specimens (figure 2*e*) was similar to the wing bud that develops in the pocket of the latero-posterior area of T2 and T3 observed on day 6 of the last nymphal instar [31], giving rise to the tegmina and membranous wings, respectively, after the imaginal moult (figure 2*f*).

**Figure 3.**
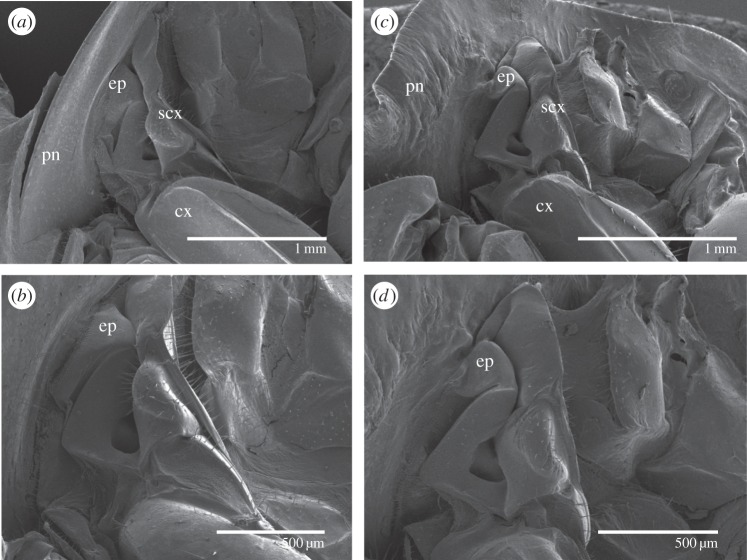
SEM examinations of the effects of *Scr* depletion in *Blattella germanica* on the prothorax pleural region. (*a*,*b*) Lateral view of T1 of a control adult female at two magnifications. (*c*,*d*) Lateral view of T1 of an adult female obtained after dsScr treatments at two magnifications. ep, epimeron; cx, coxa; scx, subcoxa; pn, hypomeral area of the pronotum.

Examination of the dorsal view of T1, T2 and T3 in controls (figure 2*g*) and in *Scr*-depleted specimens (figure 2*h*) revealed that the morphology of T1 in the latter group resembles that of T2, especially in terms of the presence of the transverse groove close to the posterior edge and the small, backward-facing, triangular protrusion in the centre of the edge in T1 of *Scr*-depleted specimens. Examination in latero-ventral view showed that the pleural area of T1 did not appear to be significantly modified in *Scr*-depleted specimens, except for the epimeron, a sclerite proximal to the subcoxal region, which was reduced and fused to the wing-like latero-posterior expansions (figure 3*a*–*d*), suggesting that this pleurite contributed to the formation of these expansions.

**Figure 4.**
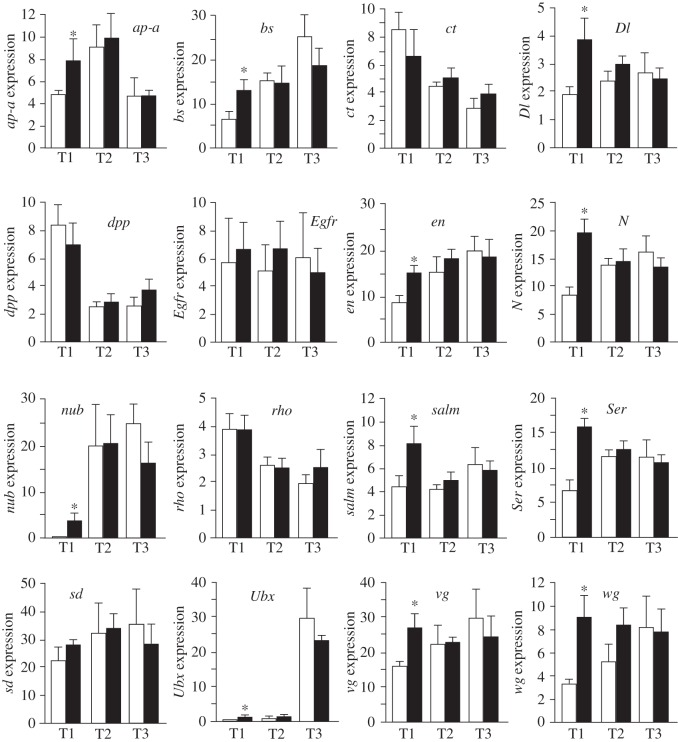
Effect of *Scr* depletion on the expression of a series of genes involved in wing development in T1, T2 and T3. *ap-a*, *apterous a*; *bs*, *blistered*; *ct*, *cut*; *Dl*, *delta*; *dpp*, *decapentaplegic*; *Egfr*, *epidermal growth factor receptor*; *en*, *engrailed*; *N*, *notch*; *nub*, *nubbin*; *rho*, *rhomboid*; *salm*, *spalt*; *Ser*, *serrate*; *sd*, *scalloped*; *Ubx*, *ultrabithorax*; *vg*, *vestigial*; *wg*, *wingless*. Expression is represented as mRNA copies per 1000 copies of actin-5c (mean ± s.e.m., *n* = 4). The asterisk indicates statistically significant differences with respect to controls (*p* < 0.05), according to REST [28].

Finally, we examined the expression levels of 16 genes involved in wing development. Interference of *Scr* resulted in a significant increase of expression of 11 of these genes in T1: *ap-a*, *bs*, *Dl*, *en*, *N*, *nub*, *Ser*, *salm*, *Ubx*, *vg* and *wg*. Only *ct*, *dpp*, *Egfr*, *rho* and *sd* were not significantly affected in T1, whereas *Scr* depletion had no significant effect on the expression of the 16 genes in T2 and T3 (figure 4). Figure S1 in the electronic supplementary material is a schematic of the relative expression of the 16 genes in control T1, T2 and T3, and in T1 from dsScr-treated specimens.

## 4. Discussion

In *B. germanica*, *Scr* depletion triggered the formation of hardened and deeply folded wing-like expansions in the latero-posterior region of T1, exactly where wing pads locate in T2 and T3 [31]. Before adult ecdysis the wing pads show a deeply folded morphology [31] very similar to that of the T1 wing-like structures formed after *Scr* depletion. The study of the expression of a number of wing-related genes in T1 wing-like structures showed that 11 out of the 16 genes studied, including *ap-a*, *nub*, *vg* and *wg*, were significantly upregulated. This indicates that at least these 11 wing-related genes are directly or indirectly repressed by *Scr* in T1. The T1 wing-like structures obtained in our experiments with *B. germanica* are similar to those reported in *P. americana*, after *Scr* depletion [9] and are reminiscent of those characterizing the Prowing (Pw) homeotic mutant of *B. germanica*, first described by Ross [32] and possibly associated with a breakage of chromosome 9 [33]. More recent studies [34] suggest that a number of different factors may be involved in the expression of Pw, but the characteristic phenotype suggests that the *Scr* gene may have been affected.

Besides the wing-like expansions, the general dorsal morphology of T1 in *Scr*-depleted specimens is reminiscent of T2, especially because of the occurrence of a transverse groove close to the posterior edge and a triangular protrusion in this edge, which are typical structures of T2. This resemblance of dorsal morphology between T1 of *Scr*-depleted specimens and T2 has been also observed in *P. americana* [9], as well as in *O. fasciatus*, where the formation of an ectopic scutellum (which is typical of T2) in T1 is well apparent [7,8], which suggested to the respective authors that *Scr* depletion triggered a partial transformation of T1 to T2.

In *Scr*-depleted specimens we observed that the epimeron, a sclerite located in the proximal area of the T1 pleural region, was smaller than in control specimens and fused to the latero-posterior expansion of the pronotum, which formed the bulk of the wing-like structures, thus suggesting that this pleurite contributed to their formation. Prothoracic latero-ventral structures were not studied in *Scr*-depleted *P. americana* [9], but in the case of *O. fasciatus* the epimeron was visibly reduced in size and the shape changed from triangular to somewhat rectangular in *Scr*-depleted specimens [7]. Similar effects have been also observed in *T. castaneum*, in which the base of the epimeron expands and fuses to the ectopic elytra formed in T1 in the adult of *Scr*-depleted specimens [26]. As pointed out by Clark-Hachtel *et al*. [26], the contribution of pleural structures to the formation of the ectopic wing in T1 of *Scr*-depleted *T. castaneum* clearly favoured the hypothesis that insect wings have a dual (tergal and pleural) origin. The results presented herein and those reported in *O. fasciatus* [7] show that the contribution of the epimeron to the formation of the T1 ectopic wing is conserved from Polyneoptera (*B. germanica*) to Condylognatha (*O. fasciatus*) and Endopterygota (*T. castaneum*), which lends further robustness to the hypothesis proposing that insect wings have a tergal and pleural origin.

It has been previously stressed [8] that in the endopterygotes *D. melanogaster* [4] and *T. castaneum* [5,6] *Scr* depletion triggers a complete homeotic transformation of T1 into T2, whereas in *O. fasciatus* [7,8] the effects mainly concern the dorsal part, including the ectopic wings, which have been considered a unique structure [8]. The present study shows that the effects of *Scr* depletion in the polyneopteran *B. germanica* are similar to those observed in the condylognathan *O. fasciatus*. Indeed, the whole data suggest that the difference between complete and partial transformation of T1 to T2 possibly derives from the different mode of metamorphosis, hemimetabolan in *B. germanica* and *O. fasciatus*, and holometabolan in *T. castaneum* and *D. melanogaster*. In the hemimetabolan mode, the juvenile nymphs already have the basic adult body plan, and the successive nymphal moults allow growth with little morphological change; the main changes occur during the imaginal moult, when wings and genitalia are completely formed [35]. Thus, the establishment of the basic adult body plan in the hemimetabolan embryo might preclude a global segment transformation in the postembryonic period, as occurs in T1 when *Scr* is interfered with, and only a partial transformation is triggered at the imaginal moult, affecting the wings and adjacent dorsal structures and, interestingly, discrete pleural sclerites associated with wing formation. Conversely, in the holometabolan mode the adult body plan is formed in the pupal stage, essentially from a series of cells destined to develop into specific adult structures, which in *D. melanogaster* are grouped into specific imaginal discs, and that are carried during the larval stages [35,36]. Under this developmental scenario, *Scr* depletion in larval stages would trigger the global transformation of T1 to T2 in the pupae.

What seems clear is that the mechanism by which the Hox gene *Scr* confers the wingless identity of T1 is conserved from cockroaches to flies. Insect wings originated in early Devonian, with the emergence of pterygotes [27]. Then, pterygotes underwent a major radiation in the Carboniferous, where a number of groups, for example within the palaeodictyopterans, meganisopterans, protorthopterans, typically had two pairs of fully developed wings in T2 and T3, and a pair of lateral lobes or winglets on the T1 tergum [1]. However, no fossils are known with fully developed wings in T1, suggesting that co-option of *Scr* to repress the formation of full wings in T1 was an early event in wing evolution. Interestingly, the firebrat *Thermobia domestica* (apterygota, Zygentoma), a primitively wingless insect, expresses *Scr* in the prothoracic dorsum [4], thus predating the emergence of specific functions of wing formation repression that operate in the pterygotes. Therefore, the tool that would facilitate the suppression of wings in T1 was already available for co-option in apterygotes.

## Supplementary Material

Figure S1: Graphical representation of the relative expression of 16 wing genes in control T1, T2 and T3, and in T1 from dsScr-treated specimens of Blattella germanica.

## Supplementary Material

Table S1. Primers used to detect transcript levels by qPCR in Blattella germanica tissues and to prepare the dsRNAs for RNAi experiments on Scr.

## Data accessibility

The sequences of all wing genes studied in this work were obtained from the *Blattella germanica* transcriptome of metanotum epidermis, available at the Gene Expression Omnibus (GEO) database (accession no. GSM1569374). In addition, the GenBank accession number of each gene is indicated in electronic supplementary material, table S1. Sequences of primers used for mRNA quantification by qRT-PCR and for dsRNA preparation are also indicated in the electronic supplementary material, table S1.
