# Supplementary material for: Tergal and pleural structures contribute to the formation of ectopic prothoracic wings in cockroaches
Source: R Soc Open Sci. 2016 Aug 3;3(8):160347. doi: 10.1098/rsos.160347 (PMC5108966; doi:10.1098/rsos.160347)
Supplement: Figure S1: Graphical representation of the relative expression of 16 wing genes in control T1, T2 and T3, and in T1 from dsScr-treated specimens of Blattella germanica. [file rsos160347supp1.pdf]

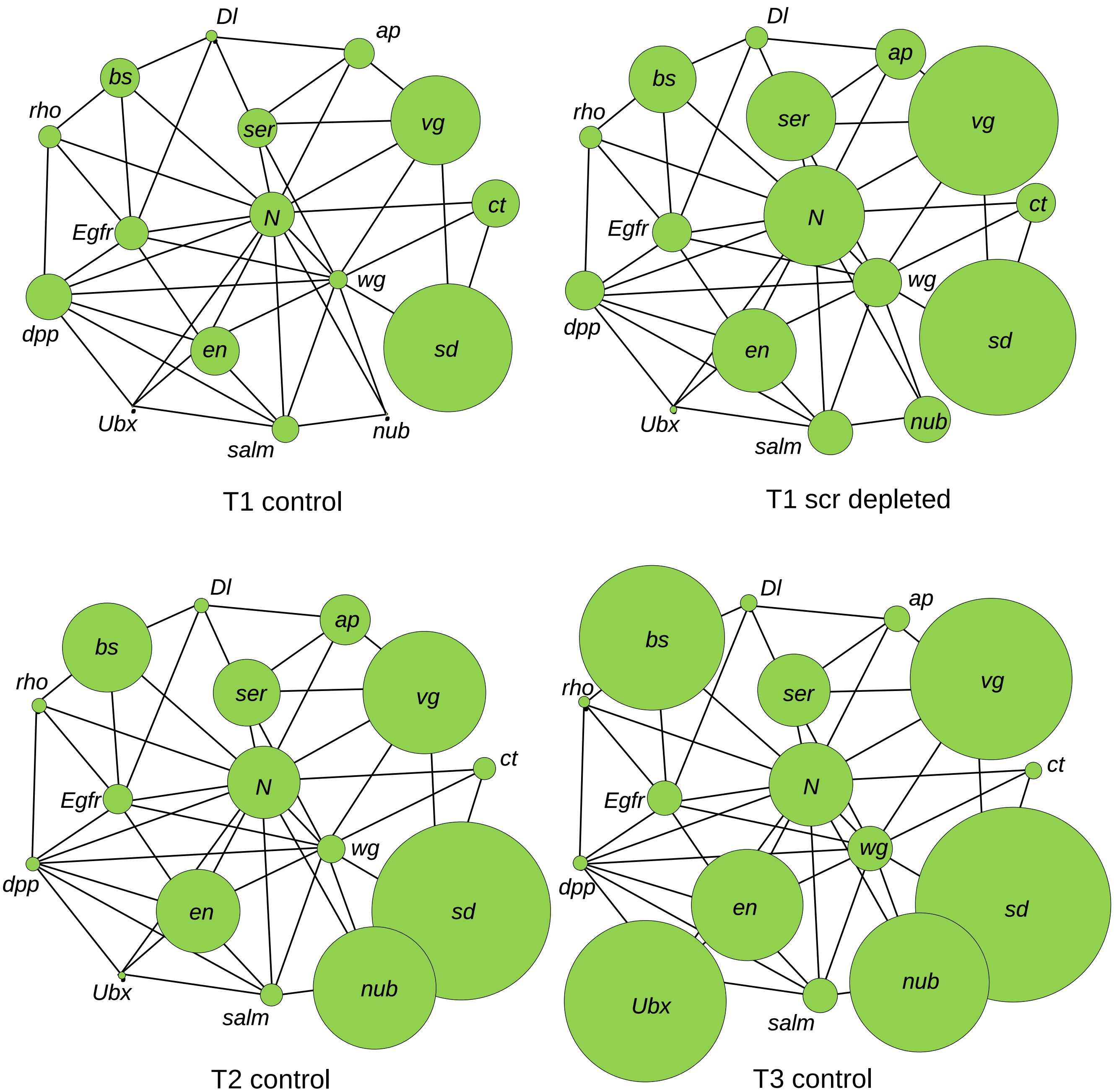

Figure S1: Graphical representation of the relative expression of the following 16 genes in control T1, T2 and T3, and in T1 from dsScr-treated specimens of *Blattella germanica*: *apterous* (*ap*), *blistered* (*bs*), *cut* (*ct*), *delta* (*Dl*), *decapentaplegic* (*dpp*), *epidermal growth factor receptor* (*Egfr*), *engrailed* (*en*), *notch* (*N*), *nubbin* (*nub*), *rhomboid* (*rho*), *scalloped* (*sd*), *serrate* (*Ser*), *spalt* (*salm*), *ultrabithorax* (*Ubx*), *vestigial* (*vg)* and *wingless* (*wg*). The diameter of the circles is proportional to the levels of expression according to data of figure 4. The interactions between genes are based on those reported for *Drosophila melanogaster* and depicted using String algorithm (http://string-db.org), although most of them have not been (yet) confirmed in *B. germanica*.
