# Supplementary material for: Tergal and pleural structures contribute to the formation of ectopic prothoracic wings in cockroaches
Source: R Soc Open Sci. 2016 Aug 3;3(8):160347. doi: 10.1098/rsos.160347 (PMC5108966; doi:10.1098/rsos.160347)
Supplement: Table S1. Primers used to detect transcript levels by qPCR in Blattella germanica tissues and to prepare the dsRNAs for RNAi experiments on Scr. [file rsos160347supp2.pdf]

Table S1. Primers used to detect transcript levels by qPCR in *Blattella germanica* tissues and to prepare the dsRNA for RNAi experiments on *Scr*. Gene abbreviations: *ap-a* (*apterous a*), *bs* (*blistered*), *ct* (*cut*), *Dl* (*delta*), *dpp* (*decapentaplegic*), *Egfr* (*epidermal growth factor receptor*), *en* (*engrailed*), *N* (*notch*), *nub* (*nubbin*), *rho* (*rhomboid*), *salm* (*spalt*), *Scr* (*sex combs reduced*), *Ser* (*serrate*), *sd* (*scalloped*), *Ubx* (*ultrabithorax*), *vg* (*vestigial*) and *wg* (*wingless*).

**Primer set**	**Forward primer (5'-3')**	**Reverse primer (5'-3')**	**Reference sequence**
**RNAi**			
Scr	CTCAGCAAGTCCCTGGTCAT	AATCGGGGGACTACTCCTTG	LT216430
**qRT-PCR**			
actin-5c	AGCTTCCTGATGGTCAGGTGA	TGTCGGCAATTCCAGGGTACATGGT	AJ862721
ap-a	CGGACTACCTGGATGTTCCG	TGGTTGATGGCGAAGTACGA	LT216431
bs	GACGGAGCTCACGTACAACA	CCAGCGGTCTTACTTTCTGC	HF912428.1
ct	AAATATGTGCTCGGCCTGTC	TGCATCTTGCGGTAACTGTC	HF969266.1
Dl	CCACTACAAGTGTTCGCCAA	TACCTCTCGCATTCGTCACA	HF969256.1
dpp	TGCCCAAAGCTTGTTGTGTG	ACAGCATGGAGATGGAGGTGA	HE965018.1
Egfr	CCAGGTACGCAATGATGAAA	ATAGGAGTGCGAGGTGGAGA	HF912429.1
en	TCTACTGCACCCGCTACTCC	GAACTCGTGCTTCAGTCGTG	LT216432
N	GCTAAGAGGCTGTTGGATGC	TGCCAGTGTTGTCCTGAGAG	HF969255.1
nub	CGTCACCAGAAGAAACAACAGA	CGAGATTGTGGTCTGTGAGAAA	LT216433
rho	TGTCCACCACCCCTATTCAT	CCCATCGCCACTGTGTAGTA	HF912427.1
salm	AACTTCTCATCCTCGTCGGC	GGCTCCGTTGCTCCACATAT	LT216434
Scr	TGGATGAAGAGGGTGCATCT	CTCAATCCTCCGTCTTCTGG	LT216430
sd	GCCCACAGAGTGCTTTCTTC	CCCCTGCCTCATCTTGAATA	HF969263.1
Ser	TCCTCTTGGCAGTGCATTTG	CTTGATCACAGAGGATGCCG	HG515375.1
Ubx	AAGAGGTCGCCAGACGTACA	TTGGAACCAAATTTTGATCTGTC	LT216435
vg	AACTGTGTGGTGTTCACTCACT	AAGGAGGGAAGTTGCGAGC	LN901335
wg	CTTGCAGGTGAAGACATGC	TCGAAGCGGTCTTTGAGGTT	HE965017.1
